# Unaltered 3’-sialyllactose and 6’-sialyllactose concentrations in human milk acutely after endurance exercise: a randomized crossover trial

**DOI:** 10.3389/fnut.2025.1638430

**Published:** 2025-10-27

**Authors:** Maëliss Cynthia Chloé Lemoine, Leesa J. Klau, Mads Holmen, Emily Rose Ashby, Finn L. Aachmann, Trygve Andreassen, Guro F. Giskeødegård, Trine Moholdt

**Affiliations:** ^1^Department of Circulation and Medical Imaging, Norwegian University of Science and Technology, Trondheim, Norway; ^2^Department of Biotechnology and Food Science, Norwegian University of Science and Technology, Trondheim, Norway; ^3^Department of Gynecology and Obstetrics, St. Olav’s Hospital, Trondheim, Norway; ^4^Central Staff, St. Olavs Hospital HF, Trondheim, Norway; ^5^Department of Public Health and Nursing, Norwegian University of Science and Technology, Trondheim, Norway; ^6^Department of Endocrinology, Clinic of Medicine, St. Olavs Hospital, Trondheim University Hospital, Trondheim, Norway

**Keywords:** breastmilk, high-intensity interval training, sialyllactose, NMR metabolomics, DREAMTIME, obesity

## Abstract

**Introduction:**

Human milk contains over 200 different types of human milk oligosaccharides (HMOs) with concentrations varying based on genetics, lifestyle, and time postpartum. Prior research indicates that exercise training during pregnancy leads to increased milk concentration of the HMO 3′-sialyllactose (3’SL), potentially improving the offspring’s metabolic development. The acute effect of postpartum exercise on HMOs concentrations in human milk is unknown. We aimed to evaluate the acute effect of moderate- and high-intensity endurance exercise on two selected sialylated HMOs in human milk.

**Methods:**

Twenty exclusively breastfeeding mothers to 6-12-weeks-old term infants were included in this randomized crossover trial. They completed three conditions in random order: no exercise (REST), moderate-intensity continuous training (MICT), and high-intensity interval training (HIIT). We collected human milk at 07:00 h, 11:00 h (immediately after rest/exercise), 12:00 h (1 h after rest/exercise) and 15:00 h (4 h after rest/exercise). Skimmed milk was analyzed by NMR spectroscopy to determine concentrations of 3’SL and 6′-sialyllactose (6’SL). We used a linear mixed model to estimate the effect of exercise on the concentrations of the selected HMOs, compared with REST.

**Results:**

All participants completed the three conditions and were included in the analyses. Exercise had no statistically significant effect on 3’SL or 6’SL concentrations. The largest mean differences in 3’SL concentrations were observed immediately after rest/exercise: MICT yielded an increase of 10% (95% confidence interval (CI) -5 to 24%, *p* = 0.19) and HIIT of 4% (95% CI -10 to 19%, *p* = 0.55). Similarly, we observed the largest mean differences in 6’SL concentration immediately after MICT, with an increase of 5% (95% CI -6 to 15%, *p* = 0.40), whereas the greatest mean difference in 6’SL concentration was seen 1 h after HIIT (6%, 95% CI -5 to 16%, *p* = 0.31). No serious adverse events occurred.

**Conclusion:**

A single endurance exercise session had no statistically significant effect on 3’SL or 6’SL concentrations in human milk. Further research should determine the effect of regular exercise training on HMO concentrations.

**Clinical trial registration:**

ClinicalTrials.gov, NCT05042414.

## 1 Introduction

As the gold standard of infant nutrition (1), human milk delivers a dynamic combination of nutrients and bioactive molecules influenced by both genetics and lifestyle factors, such as nutrition and smoking (2–4). Human milk consumption is also coterminous with the first 1,000 days of life, a critical time period to establish human metabolic mechanisms (5). The mother-to-child transmission of non-genetic factors influenced by lifestyle, which primes the infant’s development, can therefore be partly mediated by human milk composition (6). Human milk oligosaccharides (HMOs) are an important group of bioactive molecules in human milk. These non-nutritive milk components promote beneficial microbiota, protect against pathogens, and enhance immune function (7). HMOs are lactose-based molecules extended by the addition of galactose– N-acetylglucosamine disaccharides, fucose, or sialic acid residues. More than 200 distinct HMOs have been identified in human milk, with their concentrations varying across the postpartum period and between individuals (7, 8). Maternal characteristics, such as age, ethnicity, parity, and body composition affect HMOs concentration and composition (9, 10). Some studies indicate that the concentrations of some HMOs differ between mothers with obesity and those with a healthy weight, but findings are inconsistent (9–12).

The simplest sialylated oligosaccharides in human milk, 3′-sialyllactose (3′SL) and 6′-sialyllactose (6′SL), are generated in the Golgi apparatus of mammary epithelial cells when specific sialyltransferases attach sialic acid to lactose via α2-3 or α2-6 linkages. 3’SL and 6’SL promote infant gastrointestinal health and limit inflammation (13, 14). 3’SL and 6’SL supplementation also enhances exercise performance in mouse and *Cænorhabditis elegans* models, suggesting a role in cardiometabolic health (15–17). A recent Mexican study showed that mothers with overweight/obesity had lower 3’SL and 6’SL concentrations in colostrum, compared with mothers with a healthy body weight (10). The relationship between the level of these sialylated HMOs in human milk and infant growth is still unclear. Some studies report no association (18, 19), while others detected a positive correlation between 3’SL and/or 6’SL concentration and weight gain (20–22). Contrastingly, one study found a negative association between 3’SL and infant BMI (23).

The effect of maternal exercise on HMO concentrations is a largely unexplored area. In mice, exercise training during pregnancy caused increased milk concentrations of 3’SL but not 6’SL (24). The authors also showed that higher milk 3’SL concentrations mediated metabolic and cardiac health of the offspring in adulthood, with male offspring cross-fostered to trained mice displaying reduced body weight and fat percentage compared with offspring cross-fostered to sedentary mice. Based on the widespread beneficial effects of exercise on most organs and tissues in the body, it seems likely that exercise also benefits human milk composition (25). Indeed, endurance and strength training during pregnancy reduce the colostrum concentrations of two proinflammatory factors, IL-8 and TNF-*α* (26). Additionally, a single moderate-intensity exercise session in the postpartum period increased milk 12,13-diHOME, an activator of brown fat metabolism, while high-intensity endurance exercise increases milk lactate concentrations acutely after exercise (27, 28). Nevertheless, despite its major impact on metabolic health, exercise has undergone little investigation concerning its effect on human milk composition.

Our objective was to assess the acute effect of endurance exercise on 3’SL and 6’SL concentrations in human milk. Based on the noted effect of exercise on 3’SL (24), the main HMO in mice, we hypothesized that single sessions of both moderate-intensity continuous training (MICT) and high-intensity interval training (HIIT) would increase the concentration of sialylated HMOs in human milk, with the greatest effect seen after HIIT. To test our hypotheses, we analyzed 3’SL and 6’SL concentrations in human milk after a single session of MICT and HIIT in a randomized crossover study. Our results will contribute to the limited body of evidence on the impact of maternal exercise on human milk composition.

## 2 Methods

### 2.1 Study design and participants

This randomized crossover trial was undertaken at the Norwegian University of Science and Technology (NTNU) in Trondheim, Norway. The Regional Committee for Medical and Health Research Ethics, Central Norway (REK) approved the study under accession number 263493. The study is pre-registered in Clinicaltrials.gov (NCT05042414, 13/09/2021). There was no patient nor public involvement in the design, conduct or reporting of the trial. Potential harms were defined and assessed non-systematically. The trial consisted of three conditions: sitting quietly (REST), MICT, and HIIT. All participants underwent the three conditions on different days in random order, with a > 48 h washout period between any two conditions. To be eligible, participants had to be 18 years or older, 6–12 weeks port-partum after a term singleton birth, able to walk or run on a treadmill for at least 50 min, exclusively breastfeeding, and living in the Trondheim area. We excluded individuals with known cardiovascular diseases or diabetes mellitus type 1 or 2. Gestational diabetes and pre-eclampsia were not exclusion criteria. Upon arrival on the day of the first condition, we generated the sequence of the conditions for each participant using a random number generator. The computer random number generator, developed at the Faculty of Medicine and Health Science, NTNU, showed the sequence on the screen and sent it to the investigators by email. The assigned intervention remained undisclosed to participants before their arrival on subsequent test days. Due to the nature of the investigation, participants and investigators remained unblinded.

**Figure 1:**
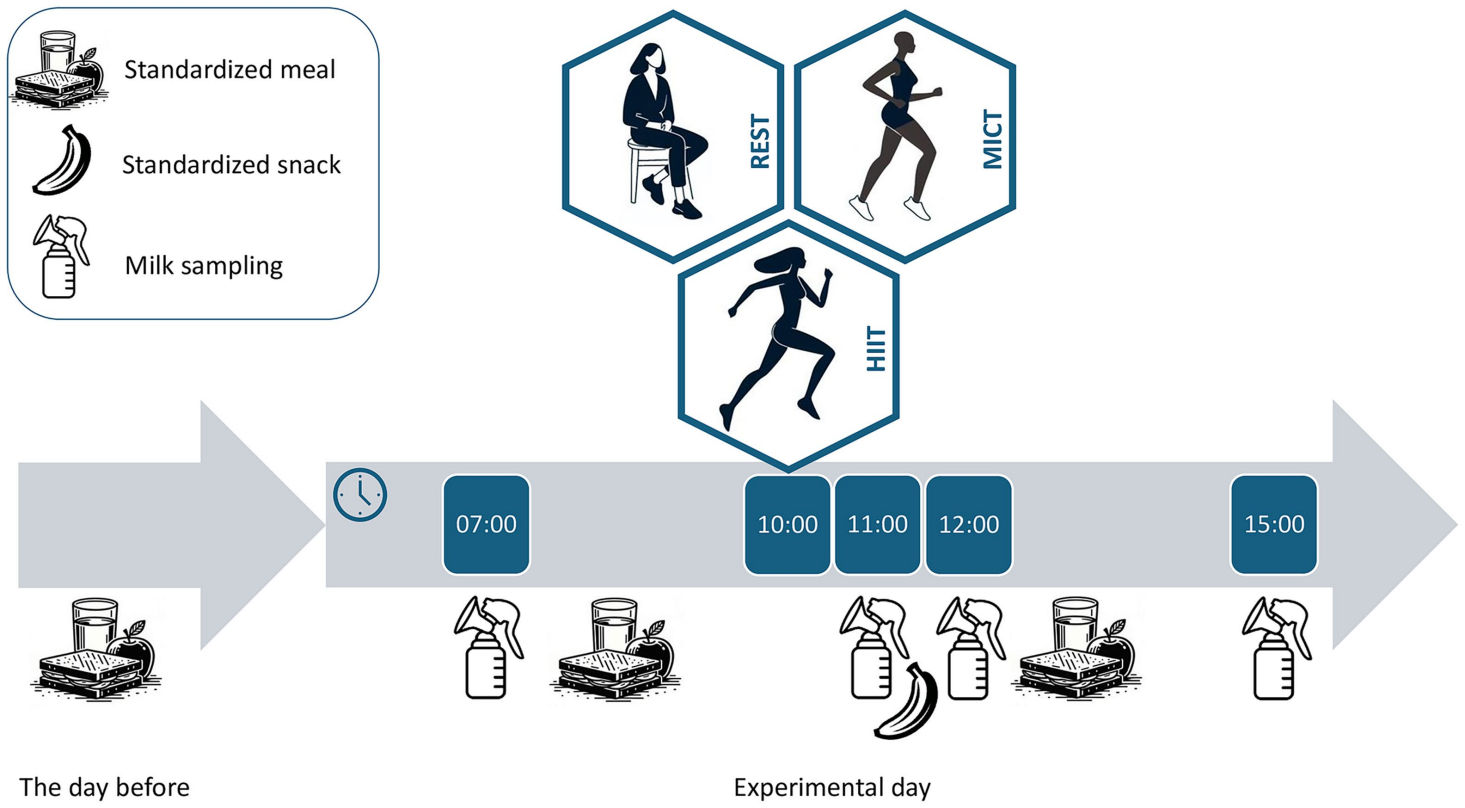
Study design. Participants consumed standardized meals and completed three conditions in random order: no exercise (REST), moderate-intensity continuous training (MICT) and high-intensity interval training (HIIT). Milk was sampled at four time points in each condition: in the morning (07:00 h) and immediately after (11:00 h), 1 h after (12:00 h), and 4 h after (15:00 h) the exercise/rest conditions.

The participants consumed standardized meals on the evening prior to the test days and on all test days to limit the effect of diet on milk composition. The type of foods, amount, and time of consumption were recorded and repeated for each test day. The participants provided four milk samples per condition: at 07:00 h (before breakfast), 11:00 h (immediately after exercise/rest), 12:00 h (1 h after exercise/rest) and 15:00 h (4 h after exercise/rest) (Figure 1). The controlled sampling times limit the potential effect of circadian variation in milk composition. The participants expressed milk using an electric breast pump (Medela Swing Flex, Medela AG, Switzerland). They collected 25 mL milk from the same breast during the whole study. At the first visit, the participants completed the International Physical Activity Questionnaire (29).

### 2.2 Exercise and rest conditions

We estimated the participants’ heart rate maximum during a preparatory visit to the laboratory prior to the test days. We defined the heart rate maximum as the highest heart rate obtained using heart rate monitors (Polar, Finland) during a maximal effort exercise test on a treadmill. We express exercise intensity for the MICT and HIIT sessions as a percentage of the heart rate maximum. MICT consisted of continuous walking/running at ~70% of heart rate maximum for 48 min. HIIT consisted of a 10-min warm-up at moderate intensity before four 4-min bouts at an intensity corresponding to 90–95% of heart rate maximum, separated by 3-min recovery periods at low-to-moderate intensity. During the exercise sessions, we recorded heart rate every fifth minute during MICT and as an average of the last 2 min of each intense bout during HIIT. During the REST condition, participants sat in a chair for 45 min. We requested the participants to refrain from exercising ≥ 48 h before test days.

### 2.3 Human milk collection and nuclear magnetic resonance spectroscopy

As described previously (30, 31), milk samples were frozen upon collection at −80 °C (samples collected at 11:00 h and 12:00 h) or at −20 °C until transport to the laboratory and storage at −80 °C (samples collected at 07:00 h and 15:00 h). Thawed samples were then skimmed by centrifugation at 10,000 x g for 60 min at 4 °C, followed by manual removal of the top fat layer and careful pipetting of the skimmed milk into a clean tube. Thereafter, the skimmed milk was frozen at −80 °C until use in the nuclear magnetic resonance (NMR) analysis.

We mixed 585 μL skimmed milk with 65 μL buffer (1.5 M KH_2_PO_4_, 2 mM NaN_3_, 0.1% 3-(trimethylsilyl)-2,2,3,3-tetradeuteropropanoic acid, pH 7.0 in D_2_O) and transferred to 5 mm NMR tubes. NMR spectra were acquired at 300 K with 128 scans on an 800 MHz Bruker Avance III HD spectrometer equipped with a 5 mm CP-TCI cryogenic probe with z-gradient using IconNMR and TopSpin 3.5.7. DREAMTIME spectra were recorded, targeting the diastereotopic protons in position 3 on each of the sialyl-groups of 3’SL and 6’SL (Supplementary Figure S3). Contrary to conventional methods, the DREAMTIME sequence enables direct quantification of the signals of interest in complex mixtures with overlapping peaks, via the selection of a unique J-coupling constant for each targeted molecule (32). Our DREAMTIME sequence required approximately 10 min per sample. 1H NOESY spectra were used as spectrum references to align DREAMTIME spectra. We determined standard addition curves based on serial dilutions of commercial 3’SL (OS04397, Biosynth Ltd., Switzerland) and 6’SL (OS04398, Biosynth Ltd., Switzerland) in skimmed milk (Supplementary Figures S1, S2). The concentrations of 3’SL and 6’SL were estimated from signal integration of DREAMTIME spectra (Supplementary Tables S1, S2) and the appropriate standard curve for each sample. Based on the high R^2^ values of the standard curves, we believe that DREAMTIME is adapted to our purpose and provided us with reliable data to calculate absolute concentrations in human milk 3’SL and 6’SL. Although some samples had concentrations outside the range for the 3’SL standard curve, we do not reach high receiver saturation effects, and we expect the linearity to hold. NMR data underwent processing and analysis using TopSpin 4.4.0 software. The DREAMTIME pulse program and a practical guide are detailed in the supporting information of the original article (32). The resonances used to select 3’SL and 6’SL signals are indicated in Supplementary Figure S3.

### 2.4 Statistical analysis

Sample size could not be formally calculated due to the fully exploratory nature of the study. The analyzed outcomes were not defined prior to study start and were exploratory. We aimed to recruit 20 participants, and all participants were included in the analyses. To assess the effect of MICT and HIIT on 3’SL and 6’SL concentrations, we used a linear mixed model with time point and the interaction between condition and time point as fixed effects and participant as random effect. Due to the randomized design, there will be no systematic differences between the baseline (07:00 h) concentrations. We have therefore not included condition as a separate effect in the model. We used a random intercept model and fitted it using restricted maximum likelihood. The REST condition and the first time point (07:00 h) served as reference. We present the estimated effect of exercise at each time point as the estimated mean difference with 95% confidence interval (CI). Both residuals and standardized residuals for 3’SL and 6’SL were checked for normality. We did not transform the data as log transformation did not improve the normality of the distribution. We also compared the 3’SL and 6’SL concentrations between participants with a healthy postpartum BMI (18.5–24.9 kg/m^2^) and those with a postpartum BMI ≥ 25.0 kg/m^2^ using a two-sample t-test with equal variances for the average values across all time points for each participant. Additionally, we explored the association between sialyllactose, adiponectin, and insulin concentrations in the milk collected at 07:00 h from our previous studies in the same cohort (30, 31) using Spearman correlation analyses. We consider *p*-values < 0.05 statistically significant. We adjusted for testing of two outcome variables by multiplying the p-value by 2 where indicated. However, we have not adjusted for multiple comparisons of time or group effects in our linear mixed model analysis as there is no consensus on how or if this should be done (33). Statistical analyses were performed using StataMP 18 (StataCorp LP). Figures were created in GraphPad Prism 10 (Dotmatics).

## 3 Results

### 3.1 Implementation of the trial and compliance with the protocol

**Figure 2:**
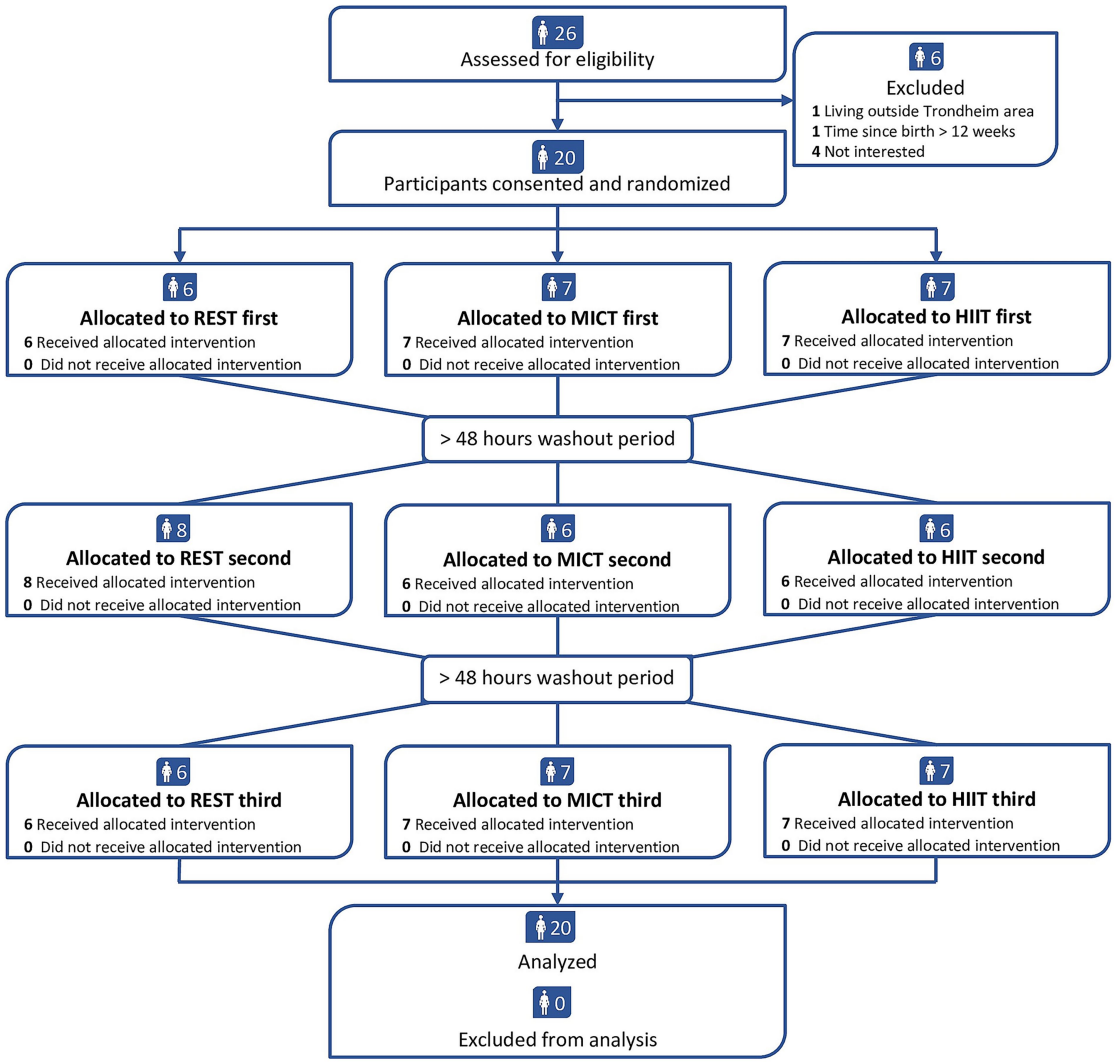
Participant flowchart. REST = no exercise, MICT = moderate-intensity continuous training, HIIT = high-intensity interval training.

We recruited 20 participants between August 2021 and May 2022. All participants completed the three conditions (Figure 2) and provided human milk at all time points, resulting in 240 milk samples. Table 1 shows the baseline characteristics of the participants. In the MICT session, the participants exercised with a mean exercise intensity of 70% (SD 1) of heart rate maximum, whereas the final 2 min of HIIT were completed at a mean 96% (SD 2) of heart rate maximum. We applied a minimum 48-h interval between REST, MICT, and HIIT in every case. The washout period averaged 7.3 days (SD 2.0 days) between the first and second condition and 6.9 days (SD 1.4 days) between the second and third condition. There were some deviations from the time points for human milk collection, with the largest deviations observed for the 15:00 h time point (average 4.7 min, SD 11.4 min). The overall time point deviation was on average 2.5 min (SD 10.1 min). We ended the recruitment of participants when we reached 20 as we had pre-specified that we aimed to include this number of participants. There were no adverse events.

**Table 1:** Baseline characteristics of participants, by which condition they completed first.

	REST (*n* = 6)	MICT (*n* = 7)	HIIT (*n* = 7)
Age, years	31 (3)	30 (2)	32 (3)
Body mass, kg	77.8 (9.6)	66.0 (6.2)	73.6 (8.1)
Body mass index, kg/m^2^	28.3 (3.8)	24.3 (3.0)	26.2 (3.3)
Fat mass, kg	28.6 (9.3)	18.1 (7.3)	24.2 (5.6)
Peak oxygen uptake, mL·kg^−1^·min^−1^	34.7 (5.0)	44.0 (7.9)	38.9 (7.8)
Time since delivery, weeks	9.1 (2.2)	8.4 (1.9)	9.3 (1.7)
Infant birth weight, g	3,963 (188)	3,338 (276)	3,700 (493)
Infant sex, female/male	4/2	6/1	3/3 (1 unknown)
Gestational diabetes, pre-eclampsia	0	0	0
Vigorous exercise in the last 7 days, min	60.0 (65.7)	92.9 (72.6)	22.1 (34.4)
Moderate exercise in the last 7 days, min	130.0 (150.2)	131.4 (89.5)	85.0 (86.5)
Walking in the last 7 days, min	340.0 (169.4)	351.4 (266.5)	525.7 (702.3)
Sitting in the last 7 days, min	2310.0 (1347.9)	2240.0 (632.3)	3780.0 (1137.4)

All participants performed all three conditions (REST, MICT, HIIT) in random order. Numbers are averages with standard deviations.

### 3.2 Human milk 3’SL and 6’SL concentrations

We detected 3’SL and 6’SL in all samples. The average 3’SL concentration for all 240 samples was 751 μmol/L, with a large variation (range: 321–1858 μmol/L). We observed 312 μmol/L as the maximal SD for 3’SL within samples from a single participant. Supplementary Table S3 shows the 3’SL concentrations at each time point during the three conditions. For 6’SL, the average concentration was 219 μmol/L (range: 58–567 μmol/L), and the maximal observed SD for a single participant 84 μmol/L (Supplementary Table S4). We found no statistically significant differences in 3’SL or 6’SL concentrations between participants with a healthy BMI versus a high BMI, but the participants with a high BMI had numerically lower 3’SL concentrations (Table 2).

**Table 2:** Comparison of 3′-sialyllactose (3’SL) and 6′-sialyllactose (6’SL) milk concentrations between individuals with a healthy BMI (18.5–24.9 kg/m^2^) and individuals with a high BMI (≥ 25 kg/m^2^).

	Healthy BMI (*n* = 9)	High BMI (*n* = 11)	*p*-value
3’SL (μmol/L)	829 (636 to 1,022)	687 (561 to 814)	0.17
6’SL (μmol/L)	225 (184 to 267)	215 (151 to 278)	0.77

Numbers are averages, with 95% confidence intervals in parenthesis. *p*-values are from two-samples *t*-tests with equal variances.

### 3.3 Acute effects of exercise on human milk sialyllactose concentrations

**Figure 3:**
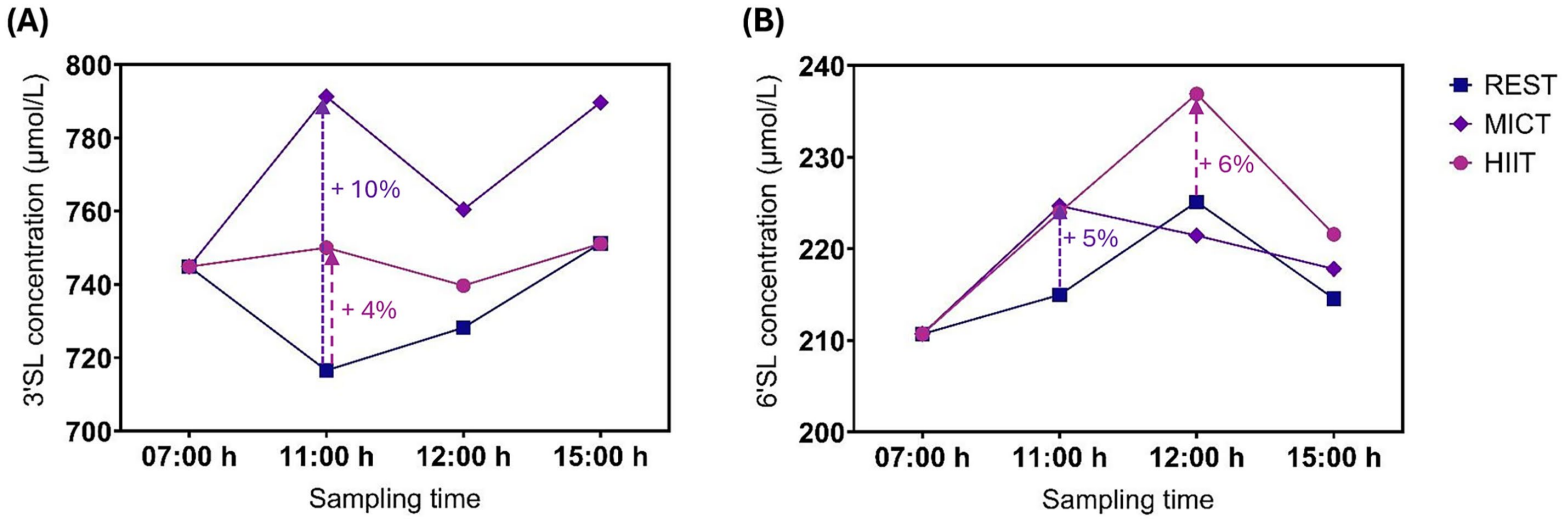
Mean 3’SL **(A)** and 6’SL **(B)** human milk concentration at different time point before and after a resting condition (REST), moderate-intensity continuous training (MICT), and high-intensity interval training (HIIT). Rest/exercise started at 10:00 h and finished before 11:00 h. Standard deviations are not represented to improve readability.

Exercise had no statistically significant effect on 3’SL or 6’SL concentrations within 4 h (Figure 3). Two individuals had a substantial decrease in 3’SL concentration of between −774 μmol/L and −1,015 μmol/L compared with the 07:00 h time point at all post-exercise time points following HIIT. We observed the largest mean differences in 3’SL concentrations immediately after exercise, with 10% (95% CI, −5 to 24%, *p* = 0.19) increase after MICT and 4% (95% CI, −10 to 19%, *p* = 0.55) increase after HIIT, compared with REST (Table 3). The largest mean differences in 6’SL concentrations were also observed immediately after exercise for MICT compared with REST, with an increase of 5% (95% CI -6 to 15%, *p* = 0.40), while for HIIT, the greatest mean difference in 6’SL concentration was an increase of 6% (95% CI -5 to 16%, *p* = 0.31) 1 h after rest/exercise (Table 4). The *p*-values above and in Tables 3, 4 are unadjusted. After adjusting for two outcome variables (3’SL and 6’SL), the p-values are between 0.38 and 1 for 3’SL and 0.62 and 1 for 6’SL.

**Table 3:** Effect of time and interactions between time and conditions on human milk 3’SL concentrations compared with samples obtained at 07:00 h.

Main effect of time				
Time point		Difference in concentration (μmol/L)	95% CI (μmol/L)	*p*-value
Immediately after exercise/rest		−28	−121 to 64	0.55
1 h after exercise/rest		−17	−109 to 76	0.72
4 h after exercise/rest		6	−86 to 99	0.89
Interaction between time and exercise				
Time point		Difference in concentration (μmol/L)	95% CI (μmol/L)	*p*-value
Immediately after exercise/rest	MICT	75	−36 to 186	0.19
	HIIT	34	−77 to 144	0.55
1 h after exercise/rest	MICT	32	−79 to 143	0.57
	HIIT	12	−99 to 122	0.84
4 h after exercise/rest	MICT	39	−72 to 149	0.50
	HIIT	0	−111 to 111	> 0.99

The interaction between time-points and moderate-intensity continuous training (MICT) and high-intensity interval training (HIIT) were compared with no exercise (REST). Data from 20 participants are estimated differences, corresponding 95% confidence interval (CI), and p-values from linear mixed model. The table shows unadjusted *p*-values. The *p*-values are between 0.38 and 1 after adjustment. The intercept was 745 μmol/L.

**Table 4:** Effect of time and interactions between time and conditions on human milk 6’SL concentrations compared with samples obtained at 07:00 h.

Main effect of time				
Time point		Difference in concentration (μmol/L)	95% CI (μmol/L)	*p*-value
Immediately after exercise/rest		4	−15 to 23	0.66
1 h after exercise/rest		14	−5 to 33	0.14
4 h after exercise/rest		4	−15 to 23	0.69
Interaction between time and exercise				
Time point		Difference in concentration (μmol/L)	95% CI (μmol/L)	*p*-value
Immediately after exercise/rest	MICT	10	−13 to 32	0.40
	HIIT	9	−14 to 32	0.44
1 h after exercise/rest	MICT	−4	−26 to 19	0.75
	HIIT	12	−11 to 34	0.31
4 h after exercise/rest	MICT	3	−19 to 26	0.78
	HIIT	7	−16 to 30	0.54

The interaction between time-points and moderate-intensity continuous training (MICT) and high-intensity interval training (HIIT) were compared with no exercise (REST). Data from 20 participants are estimated differences, corresponding 95% confidence interval (CI), and p-values from linear mixed model. The table shows unadjusted *p*-values. The *p*-values are between 0.62 and 1 after adjustment. The intercept was 211 μmol/L.

### 3.4 Exploratory associations between human milk sialyllactose, adiponectin and insulin

Adiponectin concentration showed a statistically significant association with 3’SL concentration (*p* < 0.01) but not with 6’SL concentration (*p* = 0.06) at the 07:00 timepoint. For insulin, we observed no association with 3’SL (*p* = 0.30) or 6’SL (*p* = 0.39).

## 4 Discussion

To our knowledge, this randomized crossover trial is the first study to investigate the acute effects of exercise on 3’SL and 6’SL concentrations in human milk. Contrary to our hypothesis, a single session of endurance exercise had no statistically significant effect on 3’SL or 6’SL concentrations. Future studies should investigate whether MICT transiently increases 3′SL and HIIT affects 6′SL concentrations.

Despite knowing that exercise affects the concentrations of several molecules in milk (26–28, 30), there is limited prior research on the impact of physical activity on HMO concentrations in human milk. Exercise induces profound systemic metabolic changes through increased skeletal-muscle energy demand, with outcomes depending on exercise intensity, duration, and frequency. A single bout of exercise triggers acute physiological responses, partly mediated by exercise-induced signaling molecules (exerkines) (34). Given that many metabolites, hormones, and bioactive molecules present in maternal circulation are transferred into milk, such exercise-induced changes may directly or indirectly influence human milk composition. Unlike the transient effects observed after a single exercise session, long-term adaptations arise from the cumulative impact of repeated bouts of exercise, including enhanced mitochondrial biogenesis and improved substrate utilization (35).

Although we did not observe statistically significant acute effects of endurance exercise on 3’SL or 6’SL concentrations within 4 h after exercise, Harris et al. (24) reported a positive association between physical activity during pregnancy and human milk concentrations of 3’SL at 2 months postpartum. Their findings suggest a chronic adaptation rather than an acute effect. For 6’SL, however, the relationship between human milk concentrations and physical activity is unclear. Milk 6’SL concentrations showed a weak, negative correlation with the number of daily steps in pregnancy, but no association with overall physical activity (24).

Both our findings and other studies (3, 24, 36, 37) show large inter-individual variations in human milk concentrations of 3’SL and 6’SL, making it difficult to ascertain the effect of exercise on these HMOs. We chose to define the REST condition as “sitting quietly” rather than “usual daily activities” to minimize individual variations. We also expected a higher contrast between “sitting quietly” and exercise than between “usual daily activities” and exercise. Combined with our small sample size (*n* = 20), the high inter-individual variations may explain the lack of observed significant effects in our study. We might be able to mitigate this challenge in the ongoing MILSHAKE trial, a larger randomized control trial (*n* ≈ 80) that aims to determine the chronic effects of endurance exercise training on human milk composition, including 3’SL and 6’SL (38).

Body composition is one of the factors that can impact HMO concentrations. Accordingly, another aspect of our investigation involved the association between sialylated HMOs and post-pregnancy BMI. The variations we observed in 3’SL and 6’SL concentrations between participants had an order of magnitude of 1.5 and 1, respectively. We observed a tendency of lower 3’SL concentration, but not 6’SL concentration, in mothers with overweight/obesity compared with mothers with a healthy postpartum weight. Also Harris et al. (24) found that those with a high BMI had lower 3’SL concentrations, while Urrutia-Baca et al. (10) found lower concentrations of both 3’SL and 6’SL in mothers with overweight/obesity than in those with a healthy weight.

In our cohort, 3’SL concentrations correlated significantly with adiponectin, whereas 6’SL did not show a statistically significant correlation (*p* = 0.06). On the other hand, neither 3’SL nor 6’SL correlated with insulin. The underlying mechanisms for the observed correlation between 3’SL and adiponectin should be investigated in future studies but may include links to maternal cardiorespiratory fitness. Exercise training has been shown to increase serum adiponectin concentrations in women (39) and 3’SL concentrations in human milk were found to be associated with the number of steps taken during pregnancy (24).

It is worth noting that the reported concentrations of 3’SL and 6’SL in human milk vary between studies. Some of this variation may be due to different methodologies used to determine these HMOs. Chromatography methods such as high-performance liquid chromatography, high-performance anion-exchange chromatography and liquid chromatography mass spectrometry are the most used to quantify HMOs because of their sensitivity, availability and ease-of-use. On the other hand, NMR allows for faster and more straightforward molecular quantification. The average concentrations of 3’SL (751 μmol/L) and 6’SL (219 μmol/L) in our study differed compared with what other studies covering the same lactation period have reported (36, 37, 40, 41). Two reviews including mostly studies which used chromatography methods to gather data on human milk HMO composition 31 to 100 and 15 to 90 days postpartum, calculated an average of 237 and 300 μmol/L for 3’SL and 347 and 631 μmol/L for 6’SL, respectively (40, 41). Fewer studies have used NMR to determine HMO concentrations.

Using NMR, the reported concentrations of 3’SL and 6’SL still differ between studies. One study found lower average concentrations than we did for 3’SL (110 μmol/L) and higher for 6’SL (280 μmol/L) over the first 3 months of lactation in 10 Chinese women (36). Also Smilowitz and colleagues reported lower concentrations of both HMOs 90 days postpartum compared with our results, but like us, they found higher average concentrations of 3’SL (144 μmol/L) than 6’SL (119 μmol/L) among 52 American women (37). These differences may reflect the influence of geographic location on human milk composition (42–44).

Genetic background, culture and lifestyle affect the amount and diversity of HMOs in human milk. As the concentration we found for 6’SL is similar to that found in other studies, it is possible that our Norwegian cohort presents a particularly high level of 3’SL due to genetics or lifestyle factors, such as diet. Pregnant Norwegian individuals might be less prone to consume a high-fat diet or sugar-sweetened beverages than in some other countries, two factors that have been negatively correlated with 3’SL concentration in milk (3, 45). We did not collect data on our participants’ general diet, which limits the interpretation of our results. However, the average BMI of our participants (26.2 kg/m^2^ ± 3.5) was similar to that of the participants in the study by Smilowitz and colleagues (25.3 kg/m^2^ ± 4.1), who also used NMR for HMO determination, indicating that they might share similar lifestyles (37). BMI was not reported by Kortesniemi et al. (36). Finally, the participants’ secretor status can impact at least 6’SL concentrations in human milk (37, 46). Contrary to most other studies, we did not investigate this parameter as we did not perform genetic analysis nor quantify milk fucosylation levels. Given that the Scandinavian populations (Denmark, Norway, Sweden) are genetically similar, we estimate the frequency of non-secretors to be 18% under the Hardy–Weinberg Equilibrium, based on the allele frequencies reported by NorthernSweden in the single nucleotide polymorphism database (47, 48). Genetic, cultural and lifestyle differences can impact human milk composition significantly; the results obtained from our healthy Norwegian cohort can therefore only be extrapolated to similar populations.

There is no gold-standard methodology for carrying out and analyzing NMR experiments in milk. Human milk is a complex biological fluid, explaining its poor characterization compared with other biofluids like serum or urine. Specifically, the overwhelming amount of lactose masks the signals of other milk metabolites, including peaks enabling the identification and quantification of 3’SL and 6’SL. A way to mitigate this problem is to identify all known metabolites individually and subtract the associated signals from the 1H NOESY spectra, leaving only the signals of less prominent metabolites and making it possible to quantify 3’SL and 6’SL reliably (36). This solution presents two major drawbacks: it is time-consuming and requires access to a comprehensive milk metabolite NMR library. The novel NMR sequence called DREAMTIME offers an efficient alternative to the problem of identifying specific molecules in a mixture (32). Nevertheless, a limitation of our study is that we did not analyze our samples with the more commonly used high-performance liquid chromatography coupled to mass spectrometry technique. We were thus not able to compare human milk 3’SL and 6’SL concentrations obtained with both methods. Future research should formally validate the use of DREAMTIME as an HMO quantification method, to ensure valid and reliable results.

To conclude, the present randomized crossover study demonstrated unaltered concentrations of 3’SL and 6’SL in human milk acutely after MICT and HIIT in a Norwegian cohort. The novel DREAMTIME sequence is a powerful NMR approach for analysis of complex fluids like human milk, even though further validation is required to confirm our results. This study informs health professionals and (future) parents on the relevance of a single moderate- or high-intensity exercise session in the postpartum period as a modifier for 3’SL and 6’SL milk concentrations. Further studies focusing on overweight and obese cohorts should be implemented to validate our results, as stronger effects of exercise are expected in this population compared with normal weight cohorts (49). Future research should also investigate chronic effects of exercise on human milk composition and the potential impact of such changes on infant health. Ideally, future trials will include data on habitual diet, longitudinal metabolite profiling, and infant development beyond 12 weeks postpartum.

## Data Availability

The datasets presented in this study can be found in online repositories. The names of the repository/repositories and accession number(s) can be found at: https://zenodo.org/records/15132225, 10.5281/zenodo.15132225.
