# Supplementary material for: Unaltered 3’-sialyllactose and 6’-sialyllactose concentrations in human milk acutely after endurance exercise: a randomized crossover trial
Source: Front Nutr. 2025 Oct 27;12:1638430. doi: 10.3389/fnut.2025.1638430 (PMC12599330; doi:10.3389/fnut.2025.1638430)
Supplement: Supplementary file 1 [file Table_1.DOCX]

Supplementary Material

**Supplementary Table S1.** Values used to build the 3’-sialyllactose (3’SL) standard curve showed in Figure S1. AUC: area under the curve; 6’SL: 6’-sialyllactose.

|  | Added standard (µmol/L) | AUC 3’SL | AUC 6’SL |
| --- | --- | --- | --- |
| Skimmed milk | 0 | 1 | 1.20 |
| 3'SL 37.5 | 36 | 1.07 | 1.26 |
| 3'SL 75 | 72 | 1.15 | 1.27 |
| 3'SL 150 | 145 | 1.37 | 1.26 |
| 3'SL 300 | 289 | 1.61 | 1.26 |
| 3'SL 900 | 868 | 2.99 | 1.35 |
