# Supplementary material for: Unaltered 3’-sialyllactose and 6’-sialyllactose concentrations in human milk acutely after endurance exercise: a randomized crossover trial
Source: Front Nutr. 2025 Oct 27;12:1638430. doi: 10.3389/fnut.2025.1638430 (PMC12599330; doi:10.3389/fnut.2025.1638430)
Supplement: Supplementary file 2 [file Table_2.DOCX]

Supplementary Material

**Supplementary Table S2.** Values used to build the 6’-sialyllactose (6’SL) standard curve showed in Figure S2. AUC: area under the curve; 3’SL: 3’-sialyllactose.

|  | Added standard (µmol/L) | AUC 6’SL | AUC 3’SL |
| --- | --- | --- | --- |
| Skimmed milk | 0 | 1.20 | 1 |
| 6'SL 50 | 50 | 1.58 | 1.01 |
| 6'SL 100 | 99 | 1.71 | 1.17 |
| 6'SL 200 | 199 | 2.22 | 1.05 |
| 6'SL 400 | 397 | 3.23 | 1.14 |
