# Supplementary material for: Unaltered 3’-sialyllactose and 6’-sialyllactose concentrations in human milk acutely after endurance exercise: a randomized crossover trial
Source: Front Nutr. 2025 Oct 27;12:1638430. doi: 10.3389/fnut.2025.1638430 (PMC12599330; doi:10.3389/fnut.2025.1638430)
Supplement: Supplementary file 3 [file Table_3.DOCX]

Supplementary Material

**Supplementary Table S3.** Average concentration (µmol/L) of 3'-sialyllactose (3'SL) in milk before rest/exercise (07:00 h) and immediately after (11:00 h), 1 h after (12:00 h) and 4 hrs after (15:00 h) rest/exercise. The standard deviation (SD) is given in parentheses after the average. REST: resting condition; MICT: moderate-intensity continuous training; HIIT: high-intensity interval training.

|  | **07:00 h** | **11:00 h** | **12:00 h** | **15:00 h** |
| --- | --- | --- | --- | --- |
| **REST**  69 (SD: 13) days postpartum | 744.9  (SD: 287.9) | 716.5  (SD: 242.9) | 728.20  (SD: 230.4) | 751.2  (SD: 299.8) |
| **MICT**  70 (SD: 16) days postpartum |  | 791.1  (SD: 300.7) | 760.46  (SD: 228.6) | 789.7  (SD: 258.2) |
| **HIIT**  70 (SD: 14) days postpartum |  | 750.1  (SD: 296.0) | 739.68  (SD: 291.4) | 751.1  (SD: 366.2) |
