# Supplementary material for: Unaltered 3’-sialyllactose and 6’-sialyllactose concentrations in human milk acutely after endurance exercise: a randomized crossover trial
Source: Front Nutr. 2025 Oct 27;12:1638430. doi: 10.3389/fnut.2025.1638430 (PMC12599330; doi:10.3389/fnut.2025.1638430)
Supplement: Supplementary file 4 [file Table_4.DOCX]

Supplementary Material

**Supplementary Table S4.** Average concentration (µmol/L) of 6'-sialyllactose (6'SL) in milk before rest/exercise (07:00 h) and immediately after (11:00 h), 1 h after (12:00 h) and 4 hrs after (15:00 h) rest/exercise. The standard deviation (SD) is given in parentheses after the average. REST: resting condition; MICT: moderate-intensity continuous training; HIIT: high-intensity interval training.

|  | **07:00 h** | **11:00 h** | **12:00 h** | **15:00 h** |
| --- | --- | --- | --- | --- |
| **REST**  69 (SD: 13) days postpartum | 210.7  (SD: 78.2) | 215.0  (SD: 64.9) | 225.1  (SD: 78.3) | 214.6  (SD: 68.8) |
| **MICT**  70 (SD: 16) days postpartum |  | 224.7  (SD: 101.4) | 221.5  (SD: 99.8) | 217.8  (SD: 97.2) |
| **HIIT**  70 (SD: 14) days postpartum |  | 224.0  (SD: 86.2) | 236.9  (SD: 94.3) | 221.6  (SD: 84.3) |
