# Supplementary material for: Unaltered 3’-sialyllactose and 6’-sialyllactose concentrations in human milk acutely after endurance exercise: a randomized crossover trial
Source: Front Nutr. 2025 Oct 27;12:1638430. doi: 10.3389/fnut.2025.1638430 (PMC12599330; doi:10.3389/fnut.2025.1638430)
Supplement: Supplementary file 5 [file Image_1.PDF]

Supplementary Material

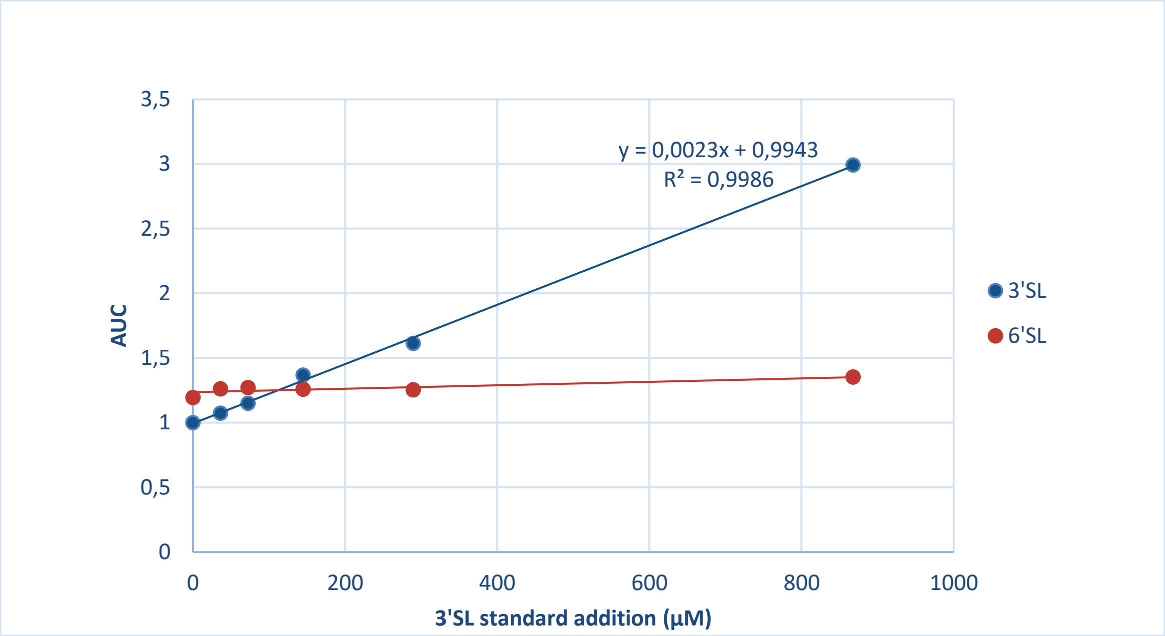

Supplementary Figure S1. Standard addition curve for 3'-sialyllactose (3'SL), based on serial dilutions of commercial 3'SL (OS04397, Biosynth Ltd, Switzerland). Added standard in µmol/L: 0, 36, 72, 145, 289, 868. Measured 6'SL is the 6'SL level naturally present in the skimmed milk used to make the standard curve; it is expected to be stable. AUC: area under the curve; 6'SL: 6'-sialyllactose.
