# Supplementary material for: Unaltered 3’-sialyllactose and 6’-sialyllactose concentrations in human milk acutely after endurance exercise: a randomized crossover trial
Source: Front Nutr. 2025 Oct 27;12:1638430. doi: 10.3389/fnut.2025.1638430 (PMC12599330; doi:10.3389/fnut.2025.1638430)
Supplement: Supplementary file 6 [file Image_2.PDF]

Supplementary Material

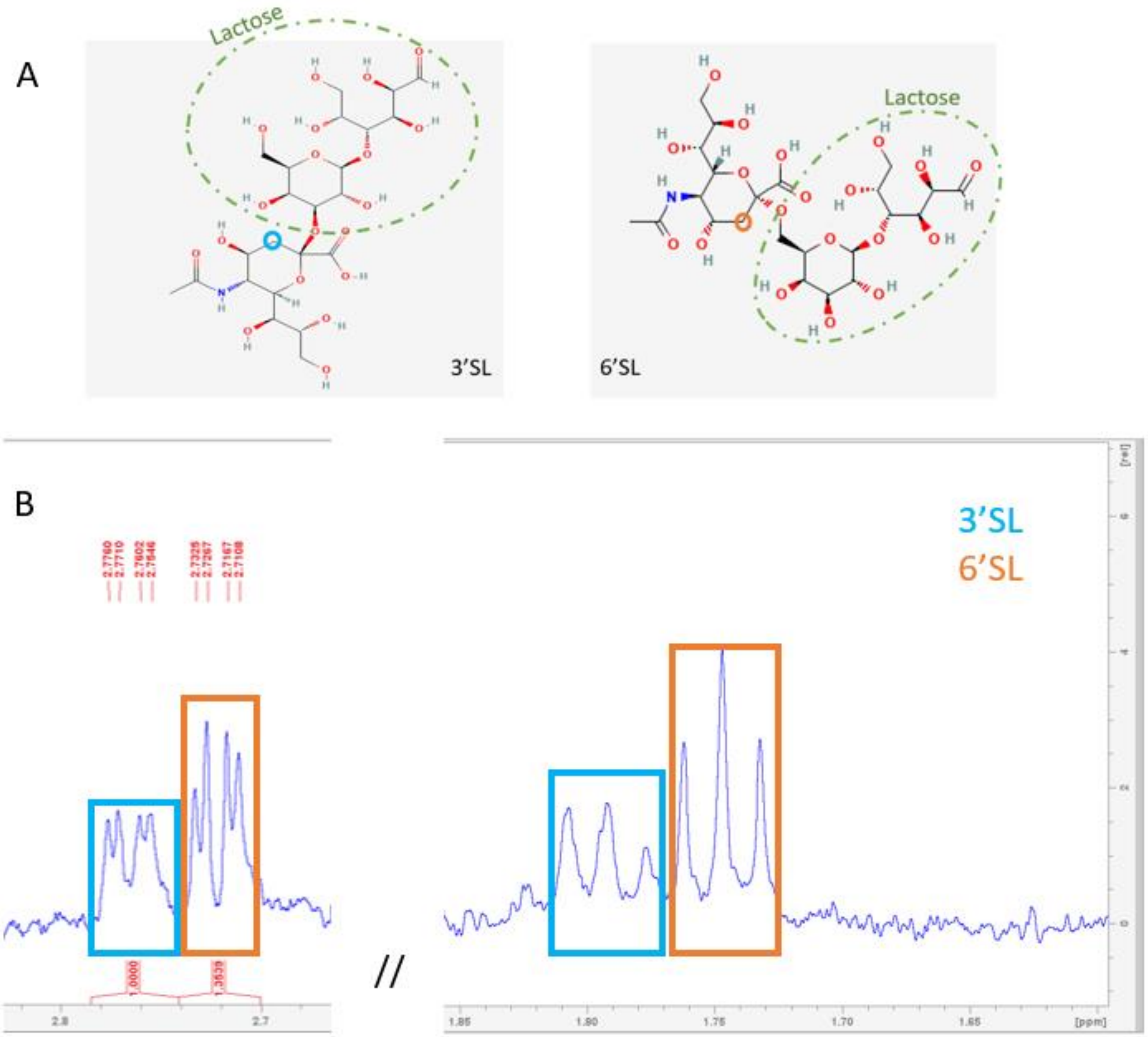

Supplementary Figure S3. Molecular structures of 3'-sialyllactose (3'SL) and 6'-sialyllactose (6'SL) **(A)**. The lactose moiety is circled with a green dotted line. For each molecule, the position of the two protons involved in the DREAMTIME analysis is indicated by a blue (3'SL) or orange (6'SL) circle. The resulting peaks are shown on the spectrum **(B)**. The signals from 3'SL (2.75 and 1.79 ppm) and 6'SL (2.71 and 1.71 ppm) are framed in blue and orange rectangles respectively. Only signals at 2.75 and 2.71 ppm were integrated and further analyzed, as they showed better separation than signals at 1.79 and 1.71 ppm. The calibration was performed on alanine doublet at 1.47 ppm from NOESY spectrum recorded prior to DREAMTIME. A compromise value for cnst11 of 10.5 Hz (^{1}H-^{1}H J coupling) provided good signal for both 3'SL and 6'SL. Other acquisition parameters: time domain (TD) = 16k, sweep width (SW) = 9.8 ppm, number of scans (ns) = 128, relaxation delay (D1)

= 2 s, and DREAMTIME waveform length (p12) = 40 ms. The spectra were recorded using water presaturation during relaxation delay. 3'SL: PubChem Identifier: CID 165618857 (https://pubchem.ncbi.nlm.nih.gov/compound/165618857#section=2D-Structure). 6'SL: PubChem Identifier: CID 643987 (https://pubchem.ncbi.nlm.nih.gov/compound/643987#section=2D-Structure)
