# Supplementary material for: Unaltered 3’-sialyllactose and 6’-sialyllactose concentrations in human milk acutely after endurance exercise: a randomized crossover trial
Source: Front Nutr. 2025 Oct 27;12:1638430. doi: 10.3389/fnut.2025.1638430 (PMC12599330; doi:10.3389/fnut.2025.1638430)
Supplement: Supplementary file 7 [file Image_3.PDF]

Supplementary Material

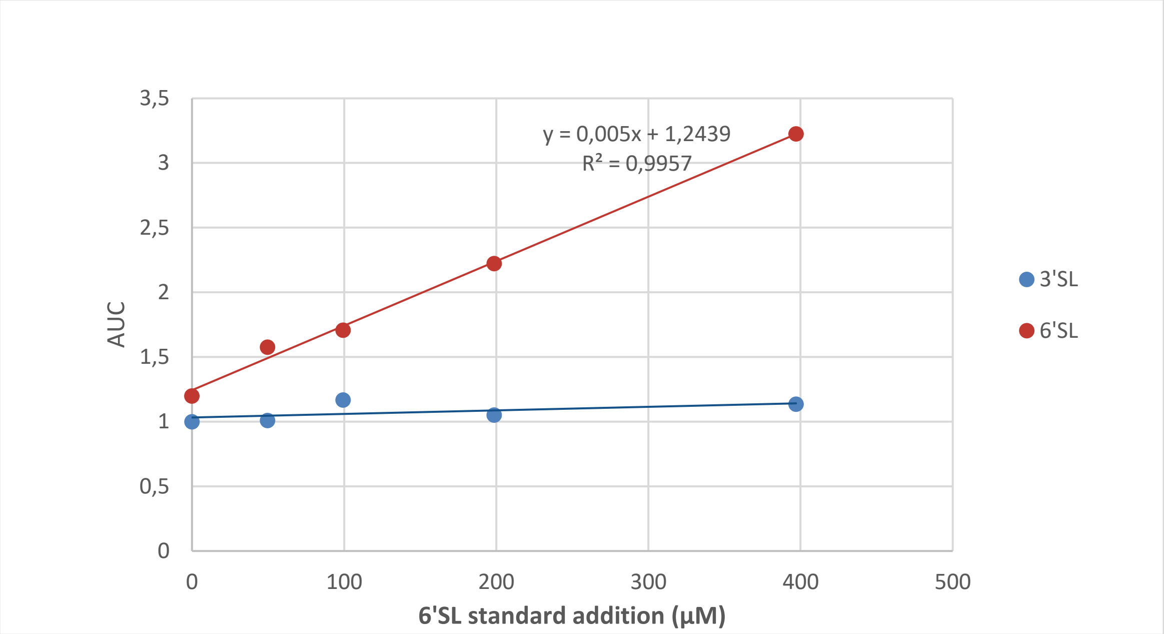

Supplementary Figure S2. Standard addition curve for 6’-sialyllactose (6’SL), based on serial dilutions of commercial 6’SL (OS04398, Biosynth Ltd, Switzerland). Added standard in µmol/L: 0, 50, 99, 199, 397. Measured 3’SL is the 3’SL level naturally present in the skimmed milk used to make the standard curve; it is expected to be stable. AUC: area under the curve; 3’SL: 3’-sialyllactose.
